# Chromene Derivatives as Selective TERRA G-Quadruplex RNA Binders with Antiproliferative Properties

**DOI:** 10.3390/ph15050548

**Published:** 2022-04-28

**Authors:** Roberta Rocca, Francesca Scionti, Matteo Nadai, Federica Moraca, Annalisa Maruca, Giosuè Costa, Raffaella Catalano, Giada Juli, Maria Teresa Di Martino, Francesco Ortuso, Stefano Alcaro, Pierosandro Tagliaferri, Pierfrancesco Tassone, Sara N. Richter, Anna Artese

**Affiliations:** 1 Department of Experimental and Clinical Medicine, Magna Graecia University of Catanzaro, Campus “Salvatore Venuta”, Viale Europa, 88100 Catanzaro, Italy; rocca@unicz.it (R.R.); giadajuli@libero.it (G.J.); teresadm@unicz.it (M.T.D.M.); tagliaferri@unicz.it (P.T.); tassone@unicz.it (P.T.); 2 Net4science Srl, Magna Graecia University of Catanzaro, 88100 Catanzaro, Italy; federica.moraca@unina.it (F.M.); maruca@unicz.it (A.M.); gscosta@unicz.it (G.C.); catalano@unicz.it (R.C.); ortuso@unicz.it (F.O.); alcaro@unicz.it (S.A.); 3 Institute for Biomedical Research and Innovation (IRIB), National Research Council of Italy (CNR), 98164 Messina, Italy; francesca.scionti@irib.cnr.it; 4 Department of Molecular Medicine, University of Padua, Via A. Gabelli 63, 35121 Padua, Italy; matteo.nadai@unipd.it; 5 Department of Pharmacy, University of Napoli Federico II, Via D. Montesano 49, 80131 Napoli, Italy; 6 Department of Health Sciences, Magna Graecia University of Catanzaro, Campus “Salvatore Venuta”, Viale Europa, 88100 Catanzaro, Italy

**Keywords:** G-quadruplex DNA, TERRA, docking, circular dichroism, mass spectrometry, biological assays

## Abstract

In mammalian cells, telomerase transcribes telomeres in large G-rich non-coding RNA, known as telomeric repeat-containing RNA (TERRA), which folds into noncanonical nucleic acid secondary structures called G-quadruplexes (G4s). Since TERRA G4 has been shown to be involved in telomere length and translation regulation, it could provide valuable insight into fundamental biological processes, such as cancer growth, and TERRA G4 binders could represent an innovative strategy for cancer treatment. In this work, the three best candidates identified in our previous virtual screening campaign on bimolecular DNA/RNA G4s were investigated on the monomolecular Tel DNA and TERRA G4s by means of molecular modelling simulations and in vitro and in cell analysis. The results obtained in this work highlighted the stabilizing power of all the three candidates on TERRA G4. In particular, the two compounds characterized by a chromene scaffold were selective TERRA G4 binders, while the compound with a naphthyridine core acted as a dual Tel/TERRA G4-binder. A biophysical investigation by circular dichroism confirmed the relative stabilization efficiency of the compounds towards TERRA and Tel G4s. The TERRA G4 stabilizing *hits* showed good antiproliferative activity against colorectal and lung adenocarcinoma cell lines. Lead optimization to increase TERRA G4 stabilization may provide new powerful tools against cancer.

## 1. Introduction

**Figure 1:**
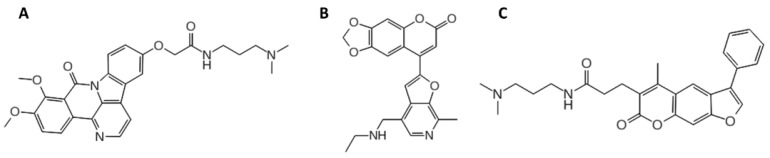
**The** 2D chemical structures of the three best *hits* found in our previous VS campaign on bimolecular Tel_2_/TERRA_2_ G4s: (**A**) *hit* **7**, characterized by a naphthyridine scaffold with a ((dimethylamino)propyl)acetamide side chain; (**B**) *hit* **15**, exhibiting a furo-chromene structure; and (**C**) *hit* **17**, distinguished by a benzofuran ring [35].

The dynamic nucleoprotein telomerase plays a central role in cellular senescence by maintaining chromosomal integrity. In particular, it adds TTAGGG sequences to the end of the chromosomes, known as telomeres, preventing chromosomal degradation or end-to-end fusion events that may cause genomic instability [1]. Telomeres are guanine-rich (G-rich) sequences, characterized by non-canonical higher-order structures called G-quadruplexes (G4s). These are characterized by two or more stacked G-tetrads that are constituted by four guanines held in a planar arrangement through a network of Hoogsteen hydrogen bonds. Moreover, further stabilization is provided by a monovalent cation coordinating the O6-lone pairs of each guanine [2]. Based on the environmental conditions, G4 can assume multiple folding topologies influenced by the number and orientation of the strands (parallel, antiparallel, and hybrid 1 and 2 types), the loop size, the groove width, and the *syn*/*anti* glycosidic bond orientation of the guanines [2,3]. For the human telomeric sequence (HTS), several G4 DNA topologies have been observed in the presence of K^+^ ions. Specifically, the parallel-stranded conformation is the only one found for the wild-type Tel_22_ AG_3_[T_2_AG_3_]_3_ crystallographic structure [4], while hybrid 1 and hybrid 2 topologies are prevalent in NMR solutions [5,6]. Conversely, antiparallel folding was revealed by NMR studies in the presence of Na^+^ [7]. Furthermore, several bimolecular and tetramolecular G4 structures can be obtained in vitro by nucleic acid sequences that include groups of contiguous guanine residues [8,9,10]. At first, telomeres were considered as transcriptionally silent regions of mammalian chromosomes, but Azzalin et al. pointed out their transcription by detecting non-coding telomeric repeat-containing RNAs (TERRA) into mammalian cells [11]. TERRA can play significant a significant role in different biological processes, such as end protection, telomeric replication, and telomerase recruitment [12,13]. Interestingly, Xu et al. demonstrated that human TERRA molecules folded in G4s similarly to the telomeric DNA and provided direct evidence about the presence of the parallel-stranded TERRA G4s in living cells, through a light-switching pyrene probe [14]. Moreover, several TERRA-binding proteins have been discovered, including telomeric duplex DNA binding proteins TRF1 and TRF2, which are Shelterin key components able to protect telomeres [15]. Balasubramanian and co-workers also demonstrated that TRF2 interacts with the G4 conformation of TERRA for binding more tightly to telomeric DNA (Tel) [16]. TRF2 promotes telomere folding by hiding the 3′-end overhang, which is not recognized as damaged DNA, thus preventing DNA damage response (DDR) activation [17]. Because the maintenance of telomeres is a key tract of cancer cells, compounds that target telomeres and their transcripts have been investigated as anticancer strategies. The therapeutic evaluation of TERRA-mediated telomerase regulation in cancer cells showed promising results. Indeed, previous studies demonstrated TERRA downregulation in advanced stages of various human cancers compared to normal tissues, suggesting that telomeric transcription is downregulated in advanced tumors [18]. Moreover, recent studies demonstrated that the identification of TERRA G4-targeting drugs induced a cytotoxic effect on high TERRA-expressing cells, where they induce a DDR at telomeres, probably by displacing TERRA from telomeres [19]. In light of this, TERRA provides a promising antitumor target as it is necessary for the formation of telomeric heterochromatin in all tumor cells, even those not expressing telomerase (ALT-positive tumors) [20]. The availability of TERRA experimental (NMR or X-ray) structures, together with a deep understanding of their topologies, is extremely helpful for the rational design of their selective ligands. Interestingly, CD, NMR, and X-ray crystallographic studies pointed out the parallel “propeller” topology as the most predominant for TERRA [21], if compared to the wide variety of G4 conformations observed for Tel [4,5,6,22,23]. Unfortunately, only the bimolecular sequence of TERRA was solved [21,24], while its unimolecular counterpart is still unavailable. While several Tel ligands have been identified and studied [25,26,27,28,29,30], few compounds have yet been proposed as TERRA binders. Among them, Collie et al. described a naphthalene diimide able to bind bimolecular TERRA with higher selectivity than BRACO-19, a ligand more selective towards Tel. This observation is suggested to be related to the presence of the 2′-OH groups in the RNA sugars, which reduce groove and loop widths, making important changes in the portion interacting with the ligand sidechains as well [31]. Therefore, the hydrogen bonds (H-bonds) network, involving the O2′ hydroxyl groups of the ribonucleotide sugars in TERRA, can be considered an important structural feature for the drug-design of selective TERRA-ligands [32]. In particular, this H-bond network tunes down the negative electrostatic surface of the target, partially explaining the observed selectivity of carboxypyridostatin (*c*PDS) [33]. A previous computational study by us pointed out that the *c*PDS high electrostatic surface, coupled to a conformational profile able to maximize the solvation contribution, is an important feature to make it selective against TERRA [34]. Starting from our previous virtual screening (VS) campaign on bimolecular telomeric DNA and RNA G4 structures (named Tel_2_ and TERRA_2_ G4, respectively) [35], in this work, the three best candidates (Figure 1) were submitted to molecular recognition studies on the 3D coordinates of monomolecular Tel and TERRA. A computational approach, based on molecular dynamics (MD) and docking simulations, was applied to target the monomolecular Tel of the 22-nt telomeric sequence 5′-AGGGTTAGGGTTAGGGTTAGGG-3′ (PDB code: 1KF1) [31] and its TERRA counterpart sequence 22-nt 5′-AGGGUUAGGGUUAGGGUUAGGG-3′. In particular, we investigated the binding affinity of the three best candidates **7**, **15**, and **17** previously discovered as dual Tel_2_ and TERRA_2_ binders [35], towards their Tel and TERRA monomolecular counterparts. With this aim, the homology model of the monomolecular TERRA was built and submitted to MD simulations to assess its geometric stability with respect to the corresponding Tel conformation. Subsequently, our docking simulations allowed us to predict the putative binding mode of all three candidates on TERRA. Furthermore, biophysical assays confirmed their stabilizing power.

## 2. Results and Discussion

### 2.1. Computational Studies

**Figure 2:**
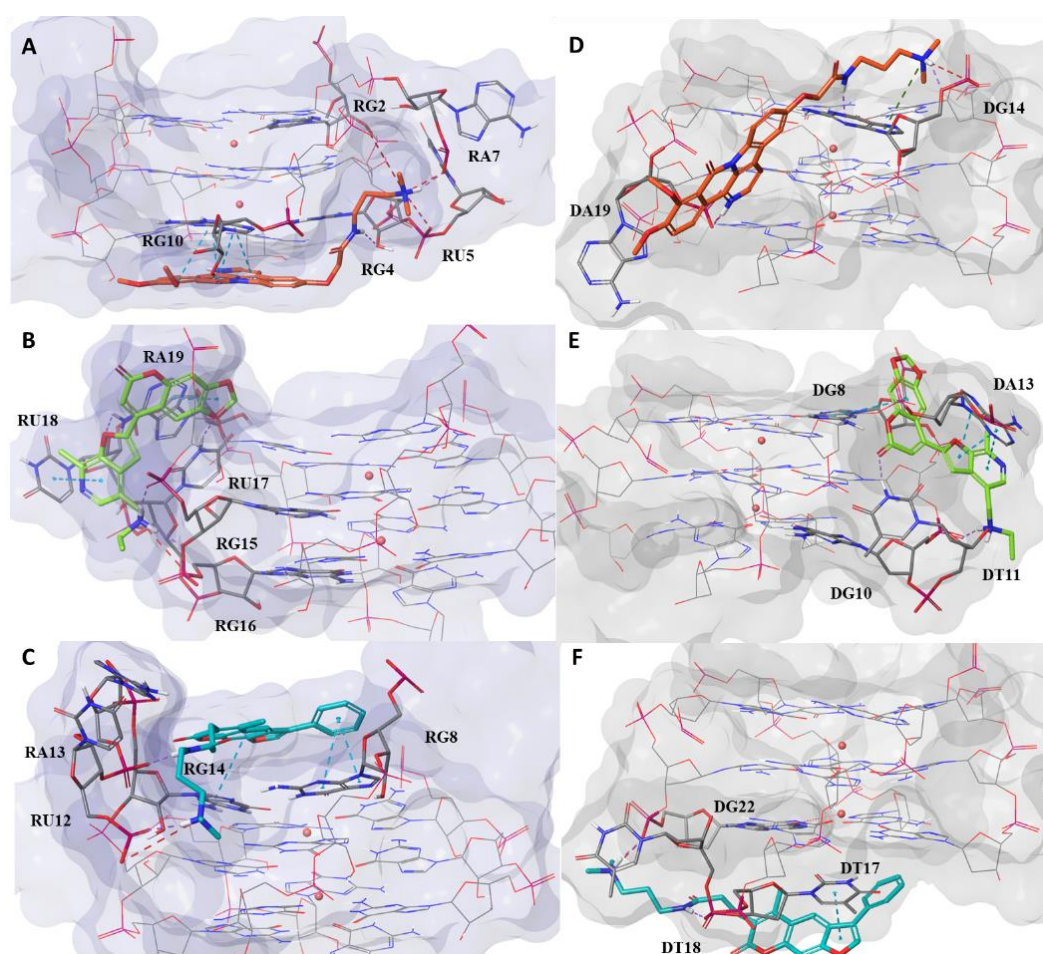
A docking pose analysis of the best thermodynamic complexes of *hits* **7** (panels (**A**,**D**)), **15** (panels (**B**,**E**)), and **17** (panels(**C**,**F**)) in complex with TERRA and Tel, respectively. For *hits* **7**, **15,** and **17,** the ligand is depicted as orange, green, and cyan carbon sticks, respectively. The nucleic acids are shown as faded blue and grey surfaces for TERRA and Tel, respectively, while the guanine residues, forming the G-tetrads, are shown as lines. Moreover, the residues interacting with the ligands are depicted as faded blue and grey carbon sticks for TERRA and Tel, respectively. K^+^ ions are represented as pink spheres. Hydrogen bonds, salt bridges, and π–π and π–cation interactions are shown as dashed violet, red, cyan, and green lines, respectively.

The binding capability of the three best *hits*, discovered in a previous VS on bimolecular Tel_2_/TERRA_2_ G4s [35], was computationally investigated on the corresponding monomolecular structures. The G4 crystallographic model of Tel (PDB ID: 1KF1) was used as template to build the corresponding TERRA G4 structure through the “*homology modelling*” approach and was submitted to MD simulations to verify the geometrical stability. MD simulations agree with the experimental data [36,37], showing higher structural stability for TERRA with respect to Tel (Supplementary Figure S1), as demonstrated by the average of the Root Mean Square deviation (RMSd) on all the heavy atoms, which is equal to 0.26 nm and 0.34 nm, respectively, for TERRA and Tel. This observation is further confirmed by the RMSd matrix (Supplementary Figure S2) calculated on the heavy atoms among all MDs conformations, from which it turns out that Tel exhibited a more significant heterogeneity than TERRA, as highlighted by the wider orange and red areas (Supplementary Figure S2A), associated with the higher RMSd values. A cluster analysis performed on all the nucleic acid heavy atoms allowed us to select the most representative conformations of both G4 targets. Specifically, to perform the subsequent docking studies, four and three conformations for Tel and TERRA, respectively, were selected, thus considering the flexibility of both targets. A visual inspection analysis of all the most representative conformations (cluster 1, 2, 3, and 4 of Tel and cluster 1, 2, and 3 of TERRA) (Supplementary Figure S3) retrieved a well-structured G-core with partial coverage of the G-tetrad at the top of the residue DA1 (Deoxyribonucleotide Adenine 1) and RA1 (Ribonucleotide Adenine 1) in Tel and TERRA, respectively. Moreover, the structure of the Tel cluster 4 was exhibited in the top position in the presence of an additional residue, DA7 (Supplementary Figure S3B). Interestingly, TERRA and Tel showed two different behaviors in the bottom position. While all TERRA representative conformations showed that the G-tetrad at the bottom position is free to interact with any end-stacking ligands, in three Tel structures (cluster 1, cluster 3, and cluster 4), we observed residue DT17 (Deoxyribonucleotide Thymine 17) interacting through stacking interactions with DG16 (Deoxyribonucleotide Guanine 16), partially preventing ligands access (Supplementary Figure S3B). The greatest structural difference between the target-selected conformations of both Tel and TERRA was instead found in the loops, where the highest heterogeneity was observed. In order to investigate the differences in the recognition of the unimolecular telomeric G4s for the three ligands, we clusterized all generated docking poses obtained against all clusters by using the angle descriptor defined by three dummy atoms, as reported in a previous work [38]. As shown in Supplementary Figure S4, *hits* **15** and **17** bound to loop in both targets, while *hit* **7** exhibited a greater number of poses positioned at the bottom of the TERRA molecule. The second favorite binding site for all compounds was the bottom position. The top position was not a favorite binding site for these compounds: we hypothesize that the presence of DA1 and RA1 in Tel and TERRA, respectively, blocks the access to the G-tetrad. In Supplementary Figure S5, we report a deeper analysis for the distribution of the binding poses to the most representative conformations of both targets. Regarding TERRA clusters, we observed heterogeneous behaviour. In fact, although all 3D structures showed that the G-tetrad in the bottom position is free to interact with the three studied compounds, as previously described, only cluster 1 and cluster 2 for *hit* **7**, and cluster 2 and cluster 1 for *hits* **15** and **17,** respectively, seem to favour this binding mode. Conversely, the lateral position appeared to be preferred in the rest of the clusters, with a higher prevalence for *hits* **15** and **17**, suggesting the conformation of the loops more favourable to accommodate a ligand. Interestingly, for cluster 4 of the Tel target, a single binding mode could be observed for all generated docking poses since all compounds bind the nucleic acid in a lateral position. In addition, Tel cluster 2 showed a preference to bind the ligands in a lateral position, especially for *hits* **7** and **17**. Finally, despite the presence at the bottom of residue DT17 that partially covers the G-tetrad, clusters 1 and 3 of all *hits* seemed to slightly prefer this binding site. Then, for each compound, we selected and deeply analyzed the energetically most stable complex with both targets (Supplementary Table S1). All three compounds showed better binding free energies when they were docked against TERRA, with ΔG_bind_ values ranging from −58.86 to −88.77 kcal/mol. Conversely, Tel complexes were characterized by ΔG_bind_ values higher than −57.96 kcal/mol. Thus, *hit* **7** potentially maintained its dual Tel/TERRA ligand profile, as previously also observed on the respective bimolecular Tel_2_/TERRA_2_ G4s [35]. Surprisingly, *hit* **15** and *hit* **17**, previously characterized as selective Tel_2_ ligands [35], turned out to have a better theoretical affinity towards the monomolecular TERRA G4. An analysis of the related single contributions of ΔG_bind_ highlighted the better contribution of Van der Waals (ΔG_bind_vdW_), lipophilic (ΔG_bind_Lipo_), and packing energy (ΔG_bind_Packing_), that is, the π–π packing correction, in TERRA complexes compared to Tel ones [39]. We next analyzed the interaction pattern of the best thermodynamic complexes (Figure 2). Specifically, *hit* **7** recognized the bottom of TERRA, confirming this binding site as the most geometrically and energetically favored (Figure 2A). The complex was stabilized by four π–π interactions, established between the naphthyridine moiety of the ligand and the nitrogenous base of the nucleobase RG10 (Ribonucleotide Guanine 10), and a π–cation between *hit* **7** pyridine ring and the potassium coordinating the last G-tetrad. This ligand also engaged three H-bonds with nucleobases RG10, RG4, and RA7 by means of the carbonyl group on the naphtyridine ring, the linear amide, and the quaternary ammonium, respectively. Its positive charge was also involved in three salt bridges with nucleobases RU5 (Ribonucleotide Uracil 5), RA7, and RG9. Conversely, in the Tel complex, *hit* **7** behaved as a loop binder by preventing the formation of the stacking interactions that stabilize its bond with TERRA (Figure 2D). This ligand interacted with Tel through three H-bonds with nucleobases DA14 and DA19 by employing its quaternary ammonium, its amide group, and its pyridinium ring, respectively. Two salt bridges were also engaged between the quaternary ammonium and the pyridinium ring of the ligand and nucleobases DA19 and DA14, respectively. *Hit* **15** (Figure 2B) appeared to form the most energetically stable complex for both G4 structures by acting as a loop binder, in agreement with the geometrical analysis of the docking binding sites. In the complex between *hit* **15** and TERRA, electrostatic contributions were the driving interactions (Supplementary Table S1). Specifically, a salt bridge, involving the quaternary ammonium of the ligand and the phosphoric group of the RG16 nucleobase, and four H-bonds were established. These latter interactions were engaged between the 1,3-dioxole portion; the furan ring; and the quaternary ammonium of the ligand and nucleobases RU17, RA19, and RG15, respectively. Conversely, in the Tel complex (Figure 2E), *hit* **15** engaged only two H-bonds between the carbonyl group of the chromene moiety and the quaternary ammonium of the ligand with nucleobases DT11 and DG10, respectively. In the complex between *hit* **15** and Tel, we observed four π–π interactions between the chromene ring and the furopyridine moiety with the nucleobases DG8 and DA13, respectively. Although only two π–π interactions were observed in the TERRA complex between the 1,3 dioxole portion and the pyridine ring of *hit* **15** with nucleobases RA19 and RU18, respectively, the ligand was able to establish several hydrophobic interactions, as highlighted by the lipophilic energetic terms in Supplementary Table S1 (ΔG_bind_Lipo_ and ΔG_bind_Packing_). Finally, the energetic analysis confirmed the behavior of loop binders for *hit* **17** when complexed to TERRA (Figure 2C). At the same time, the bottom position was disclosed as the best binding site on Tel G4 for the same compound (Figure 2F). Moreover, the best energy evaluation for the complex between *hit* **17** and TERRA G4 was related to the higher number of established favorable interactions compared to Tel. Specifically, *hit* **17** was involved in three π–π interactions and two H-bonds with TERRA G4, while in the Tel complex only one π–π interaction and one H-bond were observed. In detail, the TERRA complex showed the phenyl ring and the chromene moiety of the ligand interacting with nucleobases RG8 and RG14, respectively, through π–π interactions. Instead, the amide moiety and the quaternary ammonium engaged two H-bonds with nucleobases RA13 and RU12, respectively. Regarding the Tel complex, we observed π–π interactions between the ligand furan ring and DT17 nucleobase, while the amide portion interacted through an H-bond with DG22. Both complexes of *hit* **17** shared a salt bridge, involving its quaternary ammonium and nucleobases RU12 and DG22 for TERRA and Tel, respectively.

In a second step, we evaluated the ability of the three compounds to stabilize both TERRA and Tel. We performed 200 ns long MDs starting with thermodynamically best complexes and compared the results with respect to the stability of the related cluster conformation for each target. As shown in Supplementary Figure S6, the analysis of the RMSd trend, calculated on the heavy atoms of both targets, showed a better ability to stabilize TERRA for all compounds, compared to Tel complexes. Interestingly, *hit* **7** exhibited the best stabilizing profile on TERRA, with an average RMSd value of 0.17 nm. Moreover, it is the only compound able to form a complex with Tel showing an RMSd trend (with an average RMSd value of 0.26 nm) similar to that of the related unbound target structure (with an average RMSd value of 0.24 nm). Conversely, *hit* **17** exhibited the worst trend of RMSd on Tel (with an average RMSd value of 0.35 nm) since we observed for the entire duration of the MDs higher RMSd values compared to the unbound target, with a further increase in the last 50 ns. Regarding *hit* **15**, although in the first 100 ns of MDs it seemed to stabilize both G4 structures, in the last part of the simulation it showed a gradual increase in the RMSd trend of Tel. Finally, for each complex, the most populated structure during the MDs was selected and deeply analyzed (Supplementary Table S2 and Supplementary Figure S6). As observed in docking simulation, all the three compounds confirmed a better binding free energy if complexed with TERRA, with ΔG_bind_ values ranging from −38.36 to −85.93 kcal/mol. Conversely, Tel complexes were characterized by ΔG_bind_ values higher than −35.01 kcal/mol, except for the complex of *hit* **7**, which exhibited ΔG_bind_ value of −45.85 kcal/mol. Regarding the binding mode and the interaction pattern of the most populated structure during MDs, we observed the maintenance of the binding site only in the TERRA complexes (Supplementary Figure S7A–C). In particular, *hit* **7** always bonded the bottom portion of TERRA, as in the docking pose, and it established excellent π–π interactions between its naphthyridine moiety and nucleobases RG10 and RG16, as well as a π–cation interaction and an H-bond with RG22 and RG16, respectively. On the other hand, when comparing this structure with the initial docking pose (Figure 2A), we observed the rotation of the ligand with good interactions between its lateral chain and the first loop of TERRA. Specifically, two salt bridges were established between the quaternary ammonium of *hit* **7** and the phosphoric groups of nucleobases RA7 and RU5, while two H-bonds were observed with the sugar and the base of the RG4 (Supplementary Figure S7A). As noted in the docking pose (Figure 2B), *hit* **15** kept its lateral binding mode by establishing two π–π and a π–cation interaction with the RA19 nucleobase (Supplementary Figure S7B). Regarding *hit* **17**, the comparison of the most populated structure during MDs with the docking pose (Figure 2C) highlighted the absence of interactions between the ligand side-chain and the TERRA loop. Conversely, *hit* **17** strengthened π–π interactions between its psoralen portion and nucleobases RG8 and RG14, and it also established an additional H-bond between its carbonyl amide and RA (Supplementary Figure S7C). Interestingly, the most populated structures of MDs for Tel complexes showed a complete change in the binding site (Supplementary Figure S7D–E), except for *hit* **17**. During MDs, *hits* **7** and **15** changed their binding sites, moving from the lateral (Figure 2D–E) to the top position. The new binding mode of *hit* **7** was characterized by a strong interaction between the ligand naphthyridine moiety and nucleobase DG8, thanks to formation of six π–π, two π–cations, and one H-bond (Supplementary Figure S7D). Moreover, we also observed a π–π interaction with nucleobase DA1 and an H-bond between the ammonium group of the ligand and the sugar portion of DG14. Additionally, for *hit* **15**, we beheld several π–π interactions between its furo-chromen ring and nucleobases DG14 and DG8, but, as shown in Supplementary Figure S7E, these events caused the alteration and destabilization of the G-tetrad formed by nucleobases DG2, DG8, DG14, and DG20. Moreover, the ammonium group of the ligand was involved in two H-bonds with nucleobase DG8. Finally, the most populated structure of MDs for *hit* **17** exhibited two salt bridges between its ammonium group and the DG21 and DG22 nucleobases, an H-bond between the amide group and the DT17 and four π–π interactions with DG16 and DG10 (Supplementary Figure S7F). As noted for *hit* **15**, in this case the ligand also appeared able to induce the alteration and destabilization of the interacting G-tetrad.

### 2.2. In Vitro Analysis

**Figure 3:**
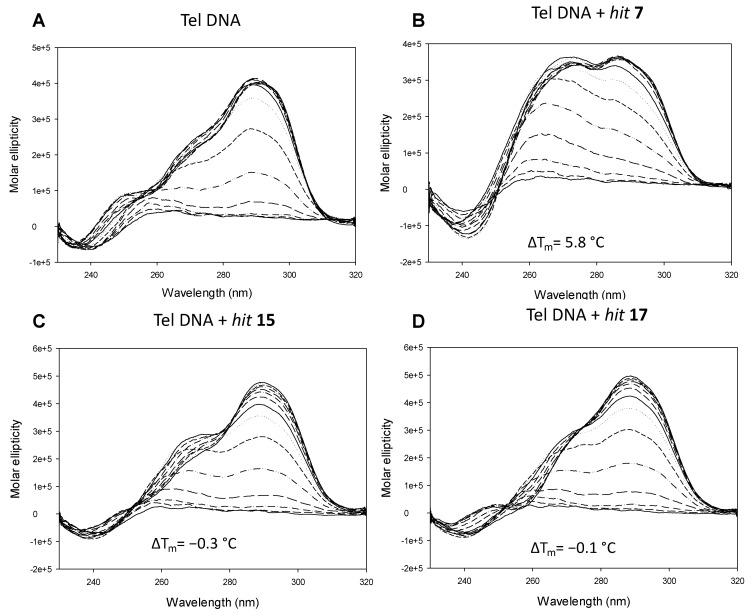
The CD thermal unfolding spectra of the nucleic acid Tel G4 4 μM in 100 mM K^+^ alone (**A**) and in the presence of the *hit* **7** 16 μM (**B**), *hit* **15** 16 μM (**C**), and *hit* **17** 16 μM (**D**).

*Hits***7**, **15,** and **17** were tested for their ability to stabilize the target Tel and TERRA G4s by means of circular dichroism (CD) melting experiments. This technique is used to obtain information about the G4 topology and stability (melting temperature, T_m_) of the G4 structured oligonucleotides. Tel G4 displayed the well-known hybrid 3 + 1 topology and a T_m_ of 67.2 ± 0.2 °C: when incubated with *hit* **7**, we observed a topological change with the appearance of a peak around 260 nm that could be ascribed to the contribution of the parallel structure, and stabilization by 5.8 °C. No significant topological changes and stabilization were observed when the target Tel G4 was incubated with *hits* **15** and **17** (Figure 3 and Figure S8).

**Figure 4:**
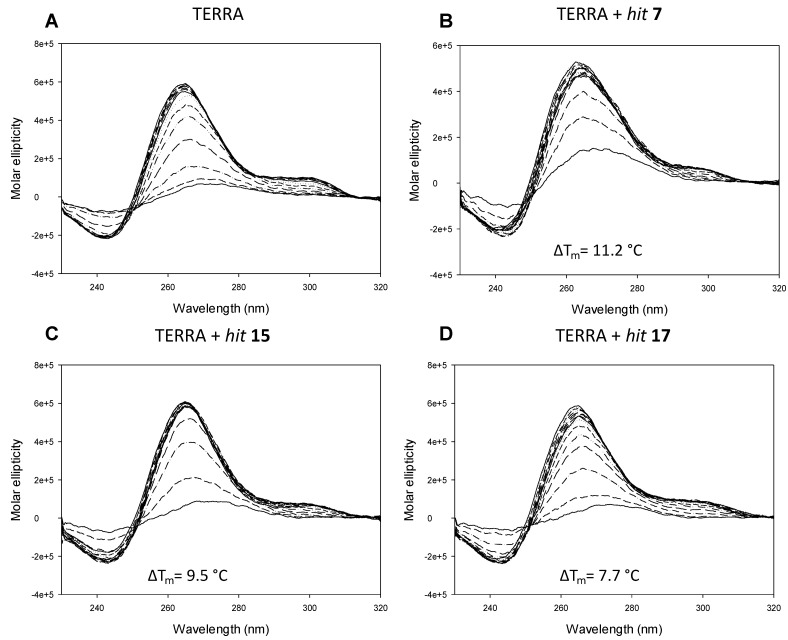
The CD thermal unfolding spectra of the nucleic acid TERRA G4 4 μM in 100 mM K^+^ alone (**A**) and in the presence of *hit* **7** 16 μM (**B**), *hit* **15** 16 μM (**C**), and *hit* **17** 16 μM (**D**).

TERRA G4 showed a prevalently parallel topology, with a maximum peak of around 260 nm and a shoulder of around 300 nm, and T_m_ of 75.2 ± 0.7 °C. Incubation with *hits* **7**, **15,** and **17** did not alter its topology but led to great stabilization of the structure, with *hit* **7** being the best stabilizer (ΔT_m_ 11.2 °C), followed by *hit* **15** (ΔT_m_ 9.5 °C) and *hit* **17** (ΔT_m_ 7.7 °C) (Figure 4 and Figure S9). These in vitro data are in perfect agreement with our MD simulations.

**Figure 5:**
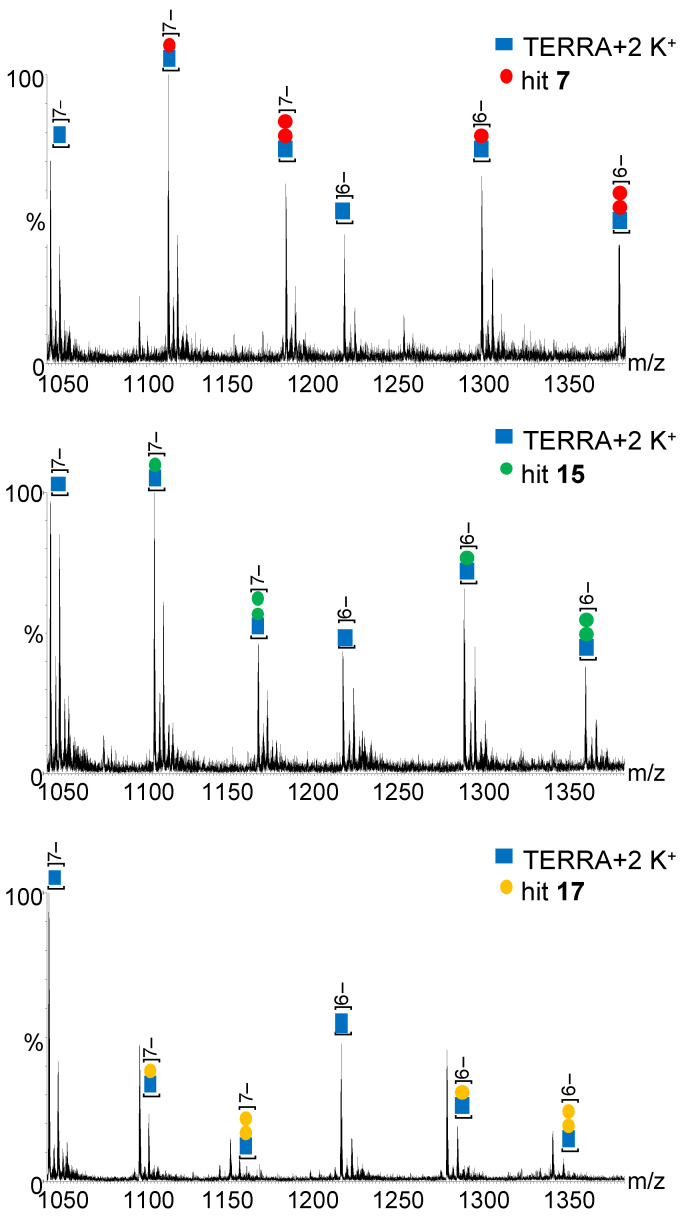
The MS spectra of TERRA (blue squares) incubated with the indicated *hits*. Samples containing TERRA oligonucleotide (5 µM) and *hit* molecule (10 μM) were incubated in MS buffer (HFIP 120 mM/TEA pH 7.4, KCl 0.8 mM, isopropanol 20%) overnight before MS analysis. A zoom on the most significant m/z range is shown. The larger m/z range is provided in Figure S11.

To measure the binding affinity of the hits to the G4-folded oligonucleotides with unmodified 5′- and 3′-ends (i.e., lacking fluorophores or biotin), we employed ESI-MS analysis, as previously described [40,41]. All *hits* displayed both a 1:1 and 1:2 binding ratio, albeit adducts with 2 bound *hit* molecules were 2–3 times less abundant than those with 1 bound *hit* molecule. For each *hit*, the binding affinity (K_D_) of the 1:1 complex with Tel and TERRA G4s was measured at three compound concentrations, corresponding to 1:1, 1:2, and 1:4 G4:compound ratios. K_D_ values were 3.4 ± 0.2 μM, 7.1 ± 1.2 μM, and 6.1 ± 0.2 μM for binding of *hits* **7**, **15,** and **17**, respectively, to TERRA G4 (Figure 5 and Figure S11). Binding affinity to Tel G4 was in general lower, with K_D_ values of 11.0 ± 0.7 μM, 18.0 ± 4.2 μM, and 22.9 ± 6.4 μM for binding of *hits* **7**, **15,** and **17**, respectively (Figure S10).

### 2.3. In Cell Assays

The cytotoxic activity of the tested compounds was evaluated on a panel of cultured human tumor cell lines: MCF7 (mammary gland adenocarcinoma), HT-29 (colorectal adenocarcinoma), and A549 (lung adenocarcinoma) cells. After 48 h treatment with the compounds, cytotoxicity was assessed by the MTT test and indicated as the concentration able to kill 50% of the cell population (CC_50_). As reported in Table 1, the colorectal adenocarcinoma HT-29 cell line was the most sensitive to compound treatment, with CC_50_ in the low micromolar range for *hits* **7** and **17** and in the nanomolar range for *hit* **15**. A549 cells had intermediate sensitivity to the *hits*, while MCF7 cells were the least affected by compounds’ treatment. In these cells, it was not possible to obtain a discrete CC_50_ value for of *hit* **7** as it exceeded the compound limit of solubility. *Hit* **15** was the most effective compound against HT-29 and A549 cells.

The sensitivity of a particular cell line to a given compound is influenced not only by the intrinsic nature of the drug but also by the characteristics of cancer, including mutations, gene expression, and copy number variation. Thus, the different response of the three cell lines observed in this study is probably due to that distinct oncogenic drivers and drug resistance mechanisms that operate in each cell line and create perturbations of the downstream pathways triggered by the compounds.

## 3. Materials and Methods

### 3.1. Target Preparation for In Silico Analysis

The crystal structure with PDB code 1KF1 and 2.1 Å resolution was selected as a tridimensional model of the parallel stranded Tel G4, featured by the 22-nt human telomeric sequence *d*[AG_3_(T_2_AG_3_)_3_] [7]. The same structure was used as a template to generate TERRA homology modeling by adding hydroxyl groups to the sugar ring. The TERRA G4 is known to be characterized by a monomorphic nature since only the parallel topology was experimentally observed [36]. K^+^ ions, coordinating the G-tetrad O6 atoms and vertically aligned in the internal G-delimited channel, were retained at their respective crystallographic positions, while all the crystallized water molecules were removed. Both Tel and TERRA structures were submitted to MD simulations, using GROMACS code ver. 4.5.1 [42]. Both nucleic acids were treated with standard *parm99* Amber force field with modified parmbsc0 [43,44] and combined with corrections ε/ζOL1 and χOL4, to improve the description of ε/ζ and χ G4 torsions, respectively [45,46,47]. For each system, the tleap module of the AmberTools program was employed to generate a topology file, which was converted into a suitable GROMACS file format using the Acpype script [48]. A truncated dodecahedron box with the TIP3P water solvent model [49] was built using periodic boundary conditions, and the global negative charge was neutralized by adding K^+^ counter-ions. To resolve bad steric contacts, both systems were energy-minimized, using 5000 steps with the steepest descent algorithm; equilibrated at 300 K through 5 ns MD under NVT conditions; and then equilibrated in the isothermal–isobaric (NPT) ensemble at 1 atm. An MD production run (200 ns) was performed in NPT, using a time step of 2 fs. The V-rescale algorithm [50] and Parrinello–Rahman barostat [51] were used to control and monitor temperature and pressure, respectively. Finally, for both targets, all conformations found during MD runs were submitted to cluster analysis with the GROMOS algorithm [52], by the g-cluster tool implemented in the GROMACS package [42]. A cut-off of 2.5 Å was used in the cluster process, with the aim to select different representative structures for the two G4 targets.

### 3.2. Molecular Docking Protocol

The most representative structures of the two G4 targets, obtained during MD runs, were used to generate grids by applying default parameters. Each energy grid was built centering the docking box on the G-tetrads centroid and setting its outer box size to 48 × 48 × 48 Å. For each docking run, 10 poses per ligand were generated and the scaling factor for the target Van der Waals radii was set to 1.0. We used the Standard Precision (SP) scoring function of Glide ver. 7.8 software of the Schrödinger suite [53] to perform docking calculations of the most promising compounds found in our previous screening on the bimolecular target [35]. The molecular structures of *hits* **7**, **15,** and **17** were previously built using the Maestro graphical user interface (*Schrödinger Release 2019: Maestro, Schrödinger, LLC., New York, NY, 2019*) [54], while their most probable protonation state at physiological pH 7.4 was computed using LigPrep (LigPrep version 2.5, *Schrödinger, LLC., New York, NY, 2012*) tool [55]. All complexes generated with the docking procedure were further submitted to the Molecular Mechanics Generalized Born/Surface Area (MM-GBSA) method [56], applying molecular mechanics and continuum solvation models, to compute their binding free energies (ΔG_bind_) [57]. The docking pose of each compound with the best ΔG_bind_ was selected and further analyzed.

### 3.3. Molecular Dynamics of the Thermodynamically Best Complexes

For each compound, the best thermodynamics complex was submitted to MD simulations, using the same MD protocol applied for both targets. To consider a comparable starting point, also the TERRA and Tel clusters that provided the best complex thermodynamics were submitted to MDs to investigate the stabilizing power of each compound. For each ligand, we calculated the electrostatic potential (ESP) by Jaguar ver. 9.3 software [58], using the 6-31G* basis set at the Hartree–Fock theory level. The restrained electrostatic potential (RESP) [59] was computed using Antechamber [60] and parameterized with General Amber Force Field (GAFF) [61]. Finally, all conformations found during MD runs were submitted to cluster analysis with the GROMOS algorithm [52], by the g-cluster tool implemented in the GROMACS package [42]. MD frames were aligned on the nucleic acid targets, and RMSd values were computed on the heavy atoms of both the target and ligand. In this case, 1.5 Å cut-off was used in the cluster process to select different representative structures of the complex for all ligands.

### 3.4. Circular Dichroism

Circular dichroism spectra were recorded on a Chirascan-Plus (Applied Photophysics, Leatherhead, UK) equipped with a Peltier temperature controller using a quartz cell of 5-mm optical path length and a scanning speed of 50 nm/min, with a response time of 4 sec over a wavelength range of 230–320 nm. The reported spectrum of each sample represents the average of 2 scans. Observed ellipticities were converted to the mean residue ellipticity (θ) = deg × cm^2^ × dmol^−1^ (molar ellipticity). Oligonucleotides were diluted from stock to the final concentration (4 μM) in the Li cacodylate buffer (10 mM, pH 7.4) with 100 mM KCl, annealed by heating at 95 °C for 5 min, and gradually cooled to room temperature. Compounds were added at 4 × G4 final concentration (16 μM). CD spectra were recorded after 24 h from 20 °C to 95 °C, with a temperature increase of 5 °C. T_m_ values were calculated according to the van’t Hoff equation, applied for a two-state transition from a folded to an unfolded state, assuming that the heat capacity of the folded and unfolded states are equal [62].

### 3.5. Binding Affinity

To determine the K_D_ of *hit* ligands to Tel and TERRA G4s, mass spectrometry (MS) analysis was performed on mixtures of oligonucleotide (5 µM) + *hit* compound (5, 10, and 20 µM). A mixture of 2 µM reference dT6 + 5 µM oligonucleotide + 5 or 10 µM *hits* was also used to check for unspecific *hit* binding. Oligonucleotides were heat denatured on MS buffer (HFIP 120 mM/TEA pH 7.4, KCl 0.8 mM, isopropanol 20%) for 5 min at 95 °C and gradually cooled to room temperature to allow the correct folding. After 4 h, *hits* were added, and samples were incubated over night at room temperature. Samples were analyzed by direct infusion electrospray ionization (ESI)-MS on a Xevo G2-XS QTOF mass spectrometer (Waters, Manchester, UK). This is a high-resolution instrument that allowed us to visualize the isotopic pattern, identify the charge state, and therefore unambiguously calculate the neutral mass of the detected species. The injection was automatically performed by an Agilent 1290 Infinity HPLC (Agilent Technologies, Santa Clara, CA, USA) equipped with an autosampler; the carrying buffer was HFIP 120 mM/TEA pH 7.4 with 20% isopropanol. A volume of 5 μL of each sample was typically injected. In all experiments, ESI source settings were electrospray capillary voltage, 1.8 kV; source and desolvation temperatures, 45 °C and 65 °C, respectively; and sampling cone voltage, 65 V. All these parameters ensured minimal DNA complex fragmentation. The instrument was calibrated using a 2 mg/mL solution of sodium iodide in 50% isopropanol. The additional use of the LockSpray during analysis provided typical <5 ppm mass accuracy. The internal standard LockSpray consisted of a solution of leu-enkephalin (1 μg/mL) in acetonitrile/water (50:50, *v*/*v*) containing 0.1% formic acid. Peak areas were used to calculate the concentration ratios, as previously reported [40,41], using the formulas:

[oligo]_free_ = C_0_ × A(oligo)^n−^/(A(oligo)^n−^ + A(oligo + *hit*)^n−^)

[oligo + *hit*] = C_0_ × A(oligo + *hit*)^n−^/(A(oligo)^n−^ + A(oligo + *hit*)^n−^)

[*hit*]_free_ = [*hit*]_tot_ − [oligo + *hit*]

K_d_ = [*hit*]_free_ × [oligo]_free_/[oligo + *hit*]

where [oligo]_free_ and [*hit*]_free_ are the concentrations of the unbound oligonucleotide and *hit*, respectively; [*hit*]_tot_ is the total concentration of *hit*; [oligo + *hit*] is the concentration of *hit* bound to oligonucleotide; C_0_ is the starting oligo (Tel or TERRA G4) concentration; A(oligo)^n−^ is the peak area of the oligonucleotide alone at charge state n^−;^ and A(oligo + *hit*)^n−^ is the peak area of *hit* bound to oligo at charge state n^−^. Peak areas were calculated using MassLynx 4.1 software (Waters), after processing steps consisting of smoothing, background subtraction, and conversion to centroid.

### 3.6. Compounds’ Cytotoxicity

Cytotoxic effects were determined by MTT assay. Compounds were dissolved and diluted into working concentrations with DMSO. All cell lines were obtained from ATCC (MCF7, human breast adenocarcinoma, cat. # HTB-22, HT-29, human colorectal adenocarcinoma, cat # HTB-38, A549, human lung carcinoma, cat # CCL-185), grown and maintained according to the manufacturer’s instructions (https://www.lgcstandards-atcc.org (accessed on 8 June 2020)). Cells were plated into 96-microwell plates to a final volume of 100 μL and allowed to attach overnight. The following day, the tested compounds were added to each well with a 0.5% final concentration of DMSO per well; each concentration was tested in triplicate. Compounds were incubated for 48 h, and control cells (without any compound but with 0.5% DMSO) were treated in the exact same conditions. Cell survival was evaluated by MTT assay: 10 μL of freshly dissolved solution of MTT (5 mg/mL in PBS) was added to each well, and after 4 h of incubation, MTT crystals were solubilized in solubilization solution (10% sodium dodecyl sulphate (SDS) and 0.01 M HCl). After overnight incubation at 37 °C, absorbance was read at 540 nm. Data were expressed as mean values of at least three experiments conducted in triplicate. The percentage of cell survival was calculated as follows: cell survival = (A_well_ − A_blank_)/(A_control_ − A_blank_) × 100, where blank denotes the medium without cells. Each experiment was repeated at least three times.

## 4. Conclusions

In this study, three compounds (*hit* **7**, *hit* **15**, and *hit* **17**), previously identified by means of a virtual screening campaign on bimolecular DNA/RNA G4s, were investigated on the monomolecular Tel DNA and TERRA G4s. Molecular docking calculations indicated all ligands were able to better recognize TERRA with respect to Tel. As previously observed on the bimolecular Tel_2_/TERRA_2_ G4s, *hit* **7** maintained its behavior as a dual Tel/TERRA ligand. Conversely, *hit* **15** and *hit* **17**, previously characterized as selective Tel_2_ ligands [35], showed better theoretical affinity towards the monomolecular TERRA G4. Moreover, MDs results highlighted that all the analyzed *hits* better stabilized TERRA G4 folding if compared to Tel, while the naphthyridine *hit* **7** confirmed its dual profile. The in vitro data corroborated our MDs since the relative *hit* stabilization efficiency on TERRA and Tel corresponded to that calculated by MDs. Analysis in cells showed that these compounds have anticancer activity. *Hit* **15**, i.e., the compound that displayed the highest selective stabilization towards TERRA, was the most active compound. Our data indicate that the tested *hits* have enhanced activity towards HT-29 cell, a model line for colorectal adenocarcinoma [63]. In particular, *hit* **15**, being over 200 times more efficient on HT-29 cell that MCF7 cells, could be a promising compound to be further optimized against colorectal cancer.

## Figures and Tables

**Table 1:** Cytotoxicity CC_50_ (μM) in human tumor cell lines measured 48 h post administration of *hits* **7**, **15,** and **17**.

	MCF7	HT-29	A549
*hit* **7**	>50	1.9 ± 0.2	20.3 ± 0.4
*hit* **15**	62.0 ± 4.1	0.3 ± 0.1	1.1 ± 0.2
*hit* **17**	28.9 ± 2.0	1.0 ± 0.1	7.9 ± 0.3

## Data Availability

Data is contained in the article and Supplementary Material.

## Supplementary Materials

The following supporting information can be downloaded at: https://www.mdpi.com/article/10.3390/ph15050548/s1, Figure S1: the plot of the RMSd values calculated on all heavy atoms during 200 ns of MDs, performed on both the parallel telomeric (Tel) DNA (black line) and TERRA (red line) G-quadruplex (G4) structures; Figure S2: the RMSd matrices calculated on the heavy atoms of all the saved structures throughout all the MDs of the parallel telomeric Tel (A) and TERRA (B) G4 structures; Figure S3: the 3D structure of all the most representative conformations of (A) TERRA and (B) Tel G4. All clusters have been superimposed, while the single cluster and the most interesting residues are shown as surface and carbon sticks, respectively. Cluster 1, cluster 2, cluster 3, and cluster 4 are reported as red, faded-blue, green, and faded-plum surface, respectively; Figure S4: a pie chart showing the distribution of all the generated docking poses of hits 7, 15, and 17, obtained against all clusters, by considering the site analysis towards both TERRA and Tel G4 targets; Figure S5: an analysis of the binding modes of hits 7, 15, and 17 on each single cluster of both G4 targets, according to the geometrical descriptors reported in a previous work [38]; Figure S6: a plot of the RMSd values calculated on all heavy atoms during 200 ns of MDs, performed on the best thermodynamic complexes of the three hits with both Tel and TERRA G4 and on the related cluster structures of both receptors. (A) An RMSd plot of TERRA cluster 2 (red line) and its related complex with hit 7 (orange line). (B) An RMSd plot of TERRA cluster 3 (red line) and its related complexes with hit 15 and hit 17 (green and cyan lines, respectively). (C) An RMSd plot of Tel cluster 4 (black line) and its related complexes with hit 7 and hit 17 (orange and cyan lines, respectively). (D) An RMSd plot of Tel cluster 1 (black line) and its related complex with hit 15 (green line); Figure S7: a binding pose analysis of the MD-generated, most populated structure of hit 7 (panels (A,D)), hit 15 (panels (B,E)), and hit 17 (panels (C,F)) in complex with TERRA and Tel, respectively. Hit 7, hit 15, and hit 17 are depicted as orange, green, and cyan carbon sticks, respectively. The nucleic acids are shown as faded blue and grey surface for TERRA and Tel, respectively, while the guanine residues, forming the G-tetrads, are shown as lines. Moreover, the residues interacting with the ligands are depicted as faded blue and grey carbon sticks for TERRA and Tel, respectively. K+ ions are represented as pink spheres. Hydrogen bonds, salt bridges, and π–π and π–cation interactions are shown as dashed violet, red, cyan, and green lines, respectively; Figure S8: the CD thermal unfolding analysis of Tel DNA G4 in complex with hits 7, 15, and 17. The melting curves of Tel G4 (4 µM) in the absence and presence of each hit (16 μM), plotted at the wavelength corresponding to the maximum CD signal; Figure S9: the CD thermal unfolding analysis of TERRA G4 in complex with hits 7, 15, and 17. The melting curves of TERRA G4 (4 µM) in the absence and presence of each hit (16 μM), plotted at the wavelength corresponding to the maximum CD signal; and Figure S10: the MS spectra of Tel (grey squares) incubated with the indicated hits. Samples containing Tel DNA oligonucleotide (5 µM) and hit molecule (10 μM) were incubated in MS buffer (HFIP 120 mM/TEA pH 7.4, KCl 0.8 mM, isopropanol 20%) overnight before MS analysis. The relevant m/z range is shown, Figure S11: the MS spectra of TERRA (blue squares) were incubated with the indicated hits. Samples containing TERRA oligonucleotide (5 µM) and hit molecule (10 μM) were incubated in MS buffer (HFIP 120 mM/TEA pH 7.4, KCl 0.8 mM, isopropanol 20%) overnight before MS analysis. The relevant m/z range is shown; Table S1: ΔGbind and related single contributions of the binding free energy for the best thermodynamic complex of hits 7, 15, and 17 with both Tel and TERRA G4. All thermodynamic values are reported in kcal/mol. In Table S1, we also reported the related cluster for each hit-target most stable complex, Table S2: ΔGbind and related single contributions of the binding free energy of the most populated cluster structure of hits 7, 15, and 17 complexed with both Tel and TERRA G4. All thermodynamic values are reported in kcal/mol.
