# Supplementary material for: Chromene Derivatives as Selective TERRA G-Quadruplex RNA Binders with Antiproliferative Properties
Source: Pharmaceuticals (Basel). 2022 Apr 28;15(5):548. doi: 10.3390/ph15050548 (PMC9147070; doi:10.3390/ph15050548)
Supplement: Supplementary file 1 [file pharmaceuticals-15-00548-s001.zip › pharmaceuticals-1582154-supplementary.pdf]

Supplementary Material for

Chromene derivatives as selective TERRA G-quadruplex RNA binders with antiproliferative properties

Roberta Rocca [1,2,†], Francesca Scionti [3,†], Matteo Nadai [4], Federica Moraca [2,5], Annalisa Maruca [2,6], Giosuè Costa [2,6], Raffaella Catalano [2,6], Giada Juli [1], Maria Teresa Di Martino [1], Francesco Ortuso [2,6], Stefano Alcaro [2,6], Pierosandro Tagliaferri [1], Pierfrancesco Tassone [1], Sara N. Richter [4,*] and Anna Artese [2,6,*]

1 Department of Experimental and Clinical Medicine, Magna Graecia University of Catanzaro, Campus "Salvatore Venuta", Viale Europa, 88100 Catanzaro, Italy; rocca@unicz.it (R.R.); giadajuli@libero.it (G.J.); teresadm@unicz.it (M.T.D.M.); tagliaferri@unicz.it (P.T.); tassone@unicz.it (P.T.)
2 Net4science Srl, Magna Graecia University of Catanzaro, 88100 Catanzaro, Italy; federica.moraca@unina.it (F.M.); maruca@unicz.it (A.M.); gscosta@unicz.it (G.C.); catalano@unicz.it (R.C.); ortuso@unicz.it (F.O.); alcaro@unicz.it (S.A.)
3 Institute for Biomedical Research and Innovation (IRIB), National Research Council of Italy (CNR), 98164 Messina, Italy; francesca.scionti@irib.cnr.it
4 Department of Molecular Medicine, University of Padua, Via A. Gabelli 63, 35121 Padua, Italy; matteo.nadai@unipd.it
5 Department of Pharmacy, University of Napoli Federico II, Via D. Montesano 49, 80131 Napoli, Italy
6 Department of Health Sciences, Magna Graecia University of Catanzaro, Campus "Salvatore Venuta", Viale Europa, 88100 Catanzaro, Italy
* Correspondence: sara.richter@unipd.it (S.N.R.); artese@unicz.it (A.A.)
† These authors contributed equally to this work.

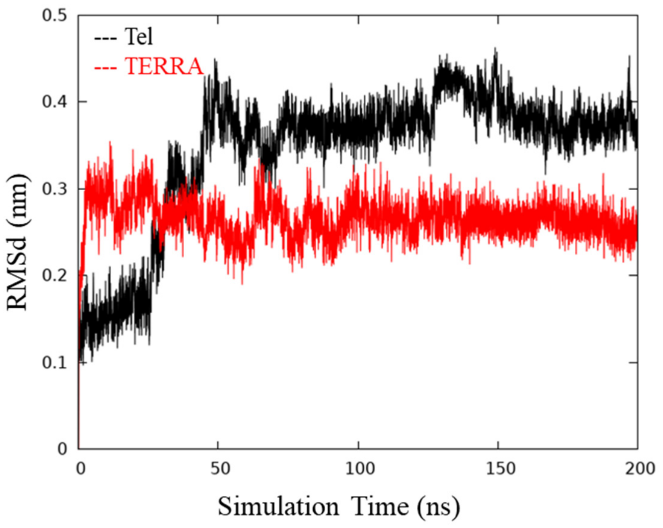

Figure S1. Plot of the RMSd values calculated on all heavy atoms during 200 ns of MDs, performed on both the parallel telomeric (Tel) DNA (black line) and TERRA (red line) G-quadruplex (G4) structures.

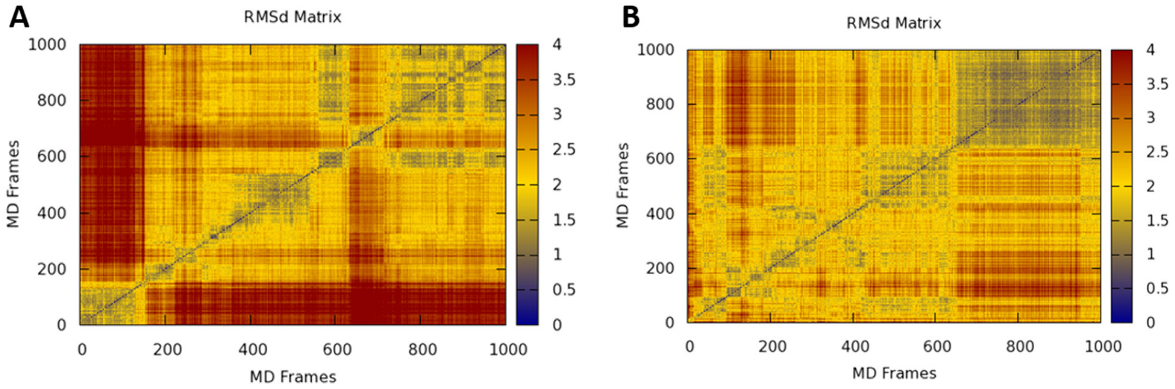

Figure S2. RMSd matrices calculated on heavy atoms of all the saved structures throughout the whole MDs of the parallel telomeric Tel **(A)** and TERRA **(B)** G4 structures.

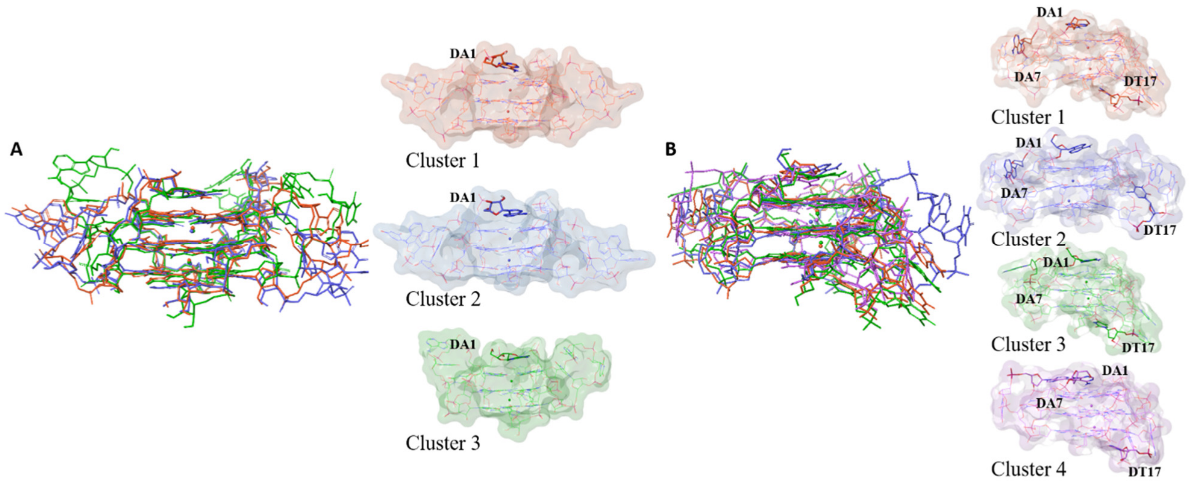

Figure S3. 3D structure of all the most representative conformation of **(A)** TERRA and **(B)** Tel G4.
All clusters have been superimposed, while the single cluster and the most interesting residues are shown as surface and carbon sticks, respectively. Cluster1, cluster2, cluster3 and cluster4 are reported as red, faded-blue, green and faded-plum surface, respectively.

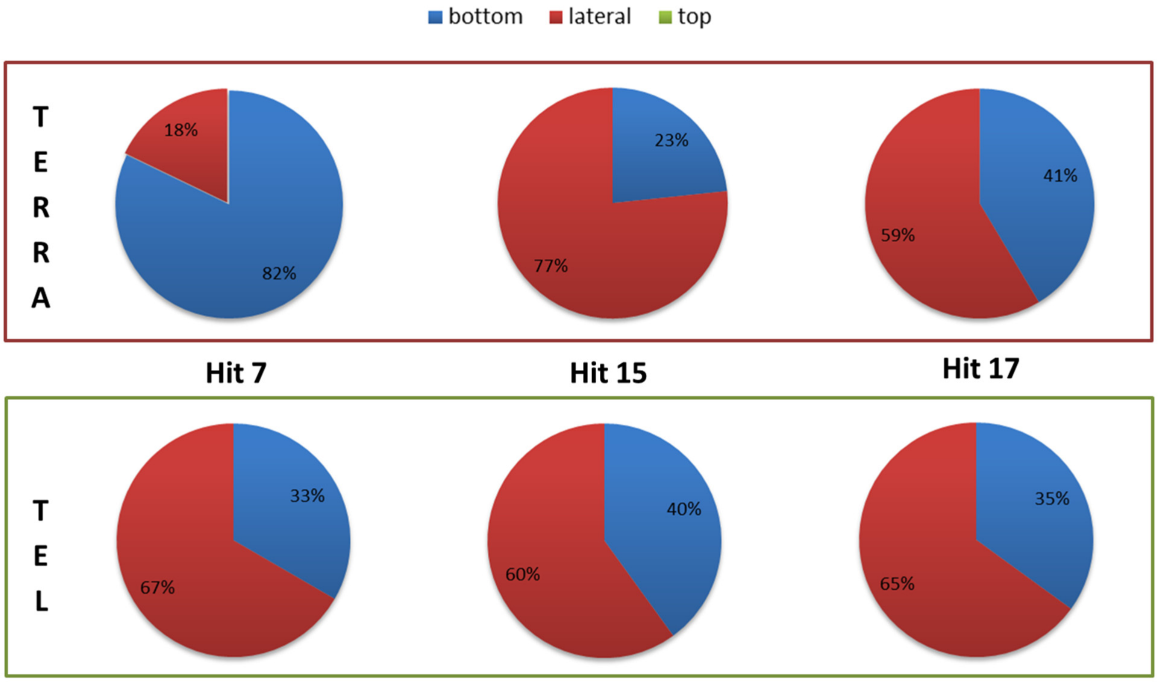

Figure S4. Pie chart showing the distribution of all the generated docking poses of *hits* **7**, **15** and **17,** obtained against all clusters, by considering the site analysis towards both TERRA and Tel G4 targets.

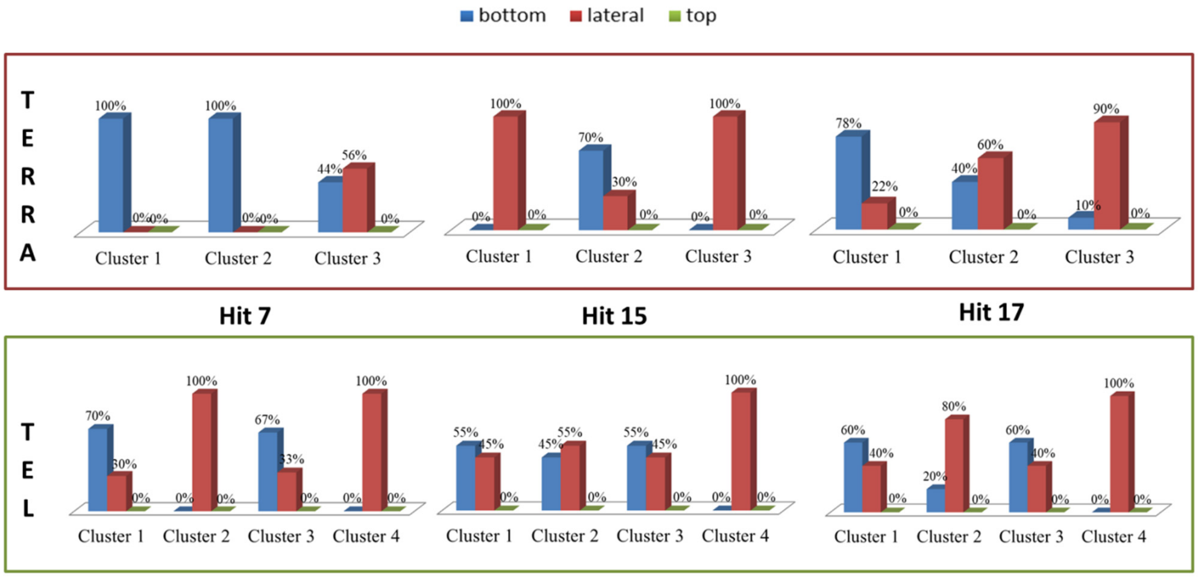

Figure S5. Analysis of the binding modes of *hits* **7**, **15** and **17** on each single cluster of both G4 targets, according to the geometrical descriptors reported in a previous work.[1]

Table S1. ΔG_{bind} and related single contributions of the binding free energy for the best thermodynamic complex of *hits* **7**, **15** and **17** with both Tel and TERRA G4. All thermodynamic values are reported in kcal/mol. In table, we also reported the related cluster for each *hit*-target most stable complex.

G4	*Hit*	Cluster	ΔG_{bind}	ΔG_{bind_Coul}	ΔG_{bind_Lipo}	$\Delta G_{bind\_Packing}$	ΔG_{bind_Solv}	ΔG_{bind_vdW}
**TERRA**	**7**	2	-88.77	-145.41	-16.58	-16.33	133.69	-44.14
	**15**	3	-58.86	-153.57	-15.67	-2.27	149.10	-36.55
	**17**	3	-64.79	-129.12	-17.68	-6.72	133.67	-44.93
**Tel**	**7**	4	-57.96	-667.19	0.00	0.00	637.36	-28.12
	**15**	1	-26.16	-86.57	0.00	0.00	95.97	-35.56
	**17**	4	-44.08	-141.77	-4.75	0.00	135.74	-33.30

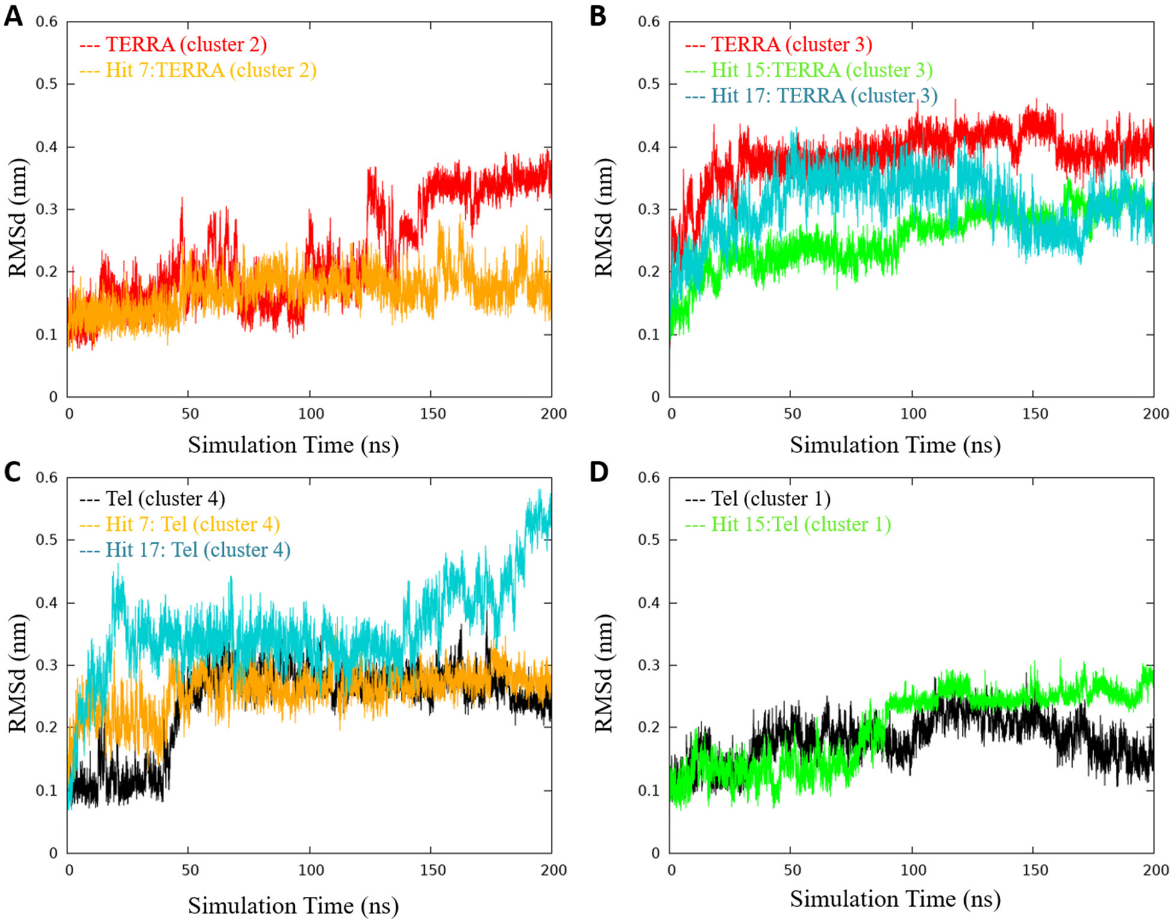

Figure S6. Plot of the RMSd values calculated on all heavy atoms during 200 ns of MDs, performed on the best thermodynamic complexes of the three *hits* with both Tel and TERRA G4 and on the related cluster structures of both receptors. **A)** RMSd plot of TERRA cluster 2 (red line) and its related complex with *hit* **7** (orange line). **B)** RMSd plot of TERRA cluster 3 (red line) and its related complexes with *hit* **15** and *hit* **17** (green and cyan lines, respectively). **C)** RMSd plot of Tel cluster 4 (black line) and its related complexes with *hit* **7** and *hit* **17** (orange and cyan lines, respectively). **D)** RMSd plot of Tel cluster 1 (black line) and its related complex with *hit* **15** (green line).

Table S2. ΔGbind and related single contributions of the binding free energy of the most populated cluster structure of *hits* **7**, **15** and **17** complexed with both Tel and TERRA G4. All thermodynamic values are reported in kcal/mol.

G4	*Hit*	ΔG_{bind}	ΔG_{bind_Coul}	ΔG_{bind_Lipo}	$\Delta G_{bind\_Packing}$	ΔG_{bind_Solv}	ΔG_{bind_vdW}
	**7**	-85.93	-133.31	-19.12	-15.20	133.25	-55.00
**TERRA**	**15**	-38.36	-96.65	-9.88	-12.47	100.70	-22.03
	**17**	-52,60	-102.84	-6.56	-12.39	115.59	-46.90
	**7**	-45.85	-561.67	-3.13	-3.30	579.78	-59.56
**Tel**	**15**	-25.15	-75.96	-0.53	0.00	90.47	-40.44
	**17**	-35.01	-136.45	-2.61	-0.02	153.18	-51.53

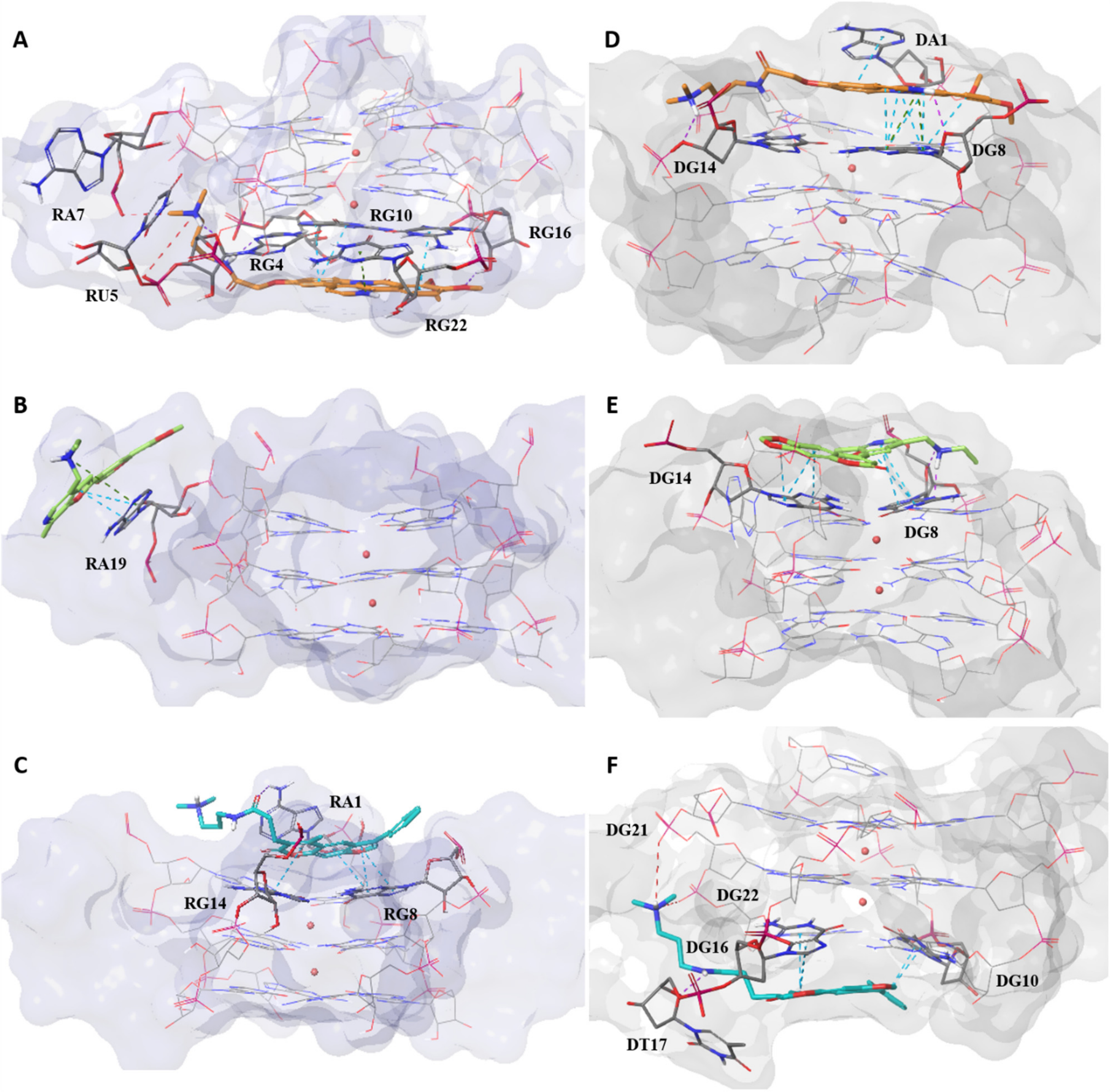

Figure S7. Binding pose analysis of the MD generated most populated structure of *hit* **7** (panels **A** and **D**), *hit* **15** (panels **B** and **E**), and *hit* **17** (panels **C** and **F**) in complex with TERRA and Tel, respectively. *Hit* **7**, *hit* **15** and *hit* **17** are depicted as

orange, green and cyan carbon sticks, respectively. The nucleic acids are shown as faded blue and grey surface for TERRA and Tel, respectively, while the guanine residues, forming the G-tetrads, are shown as lines. Moreover, the residues interacting with the ligands are depicted as faded blue and grey carbon sticks for TERRA and Tel, respectively. K^+ ions are represented as pink spheres. Hydrogen bonds, salt bridges, π-π, and π-cation interactions are shown as dashed violet, red, cyan, and green lines, respectively.

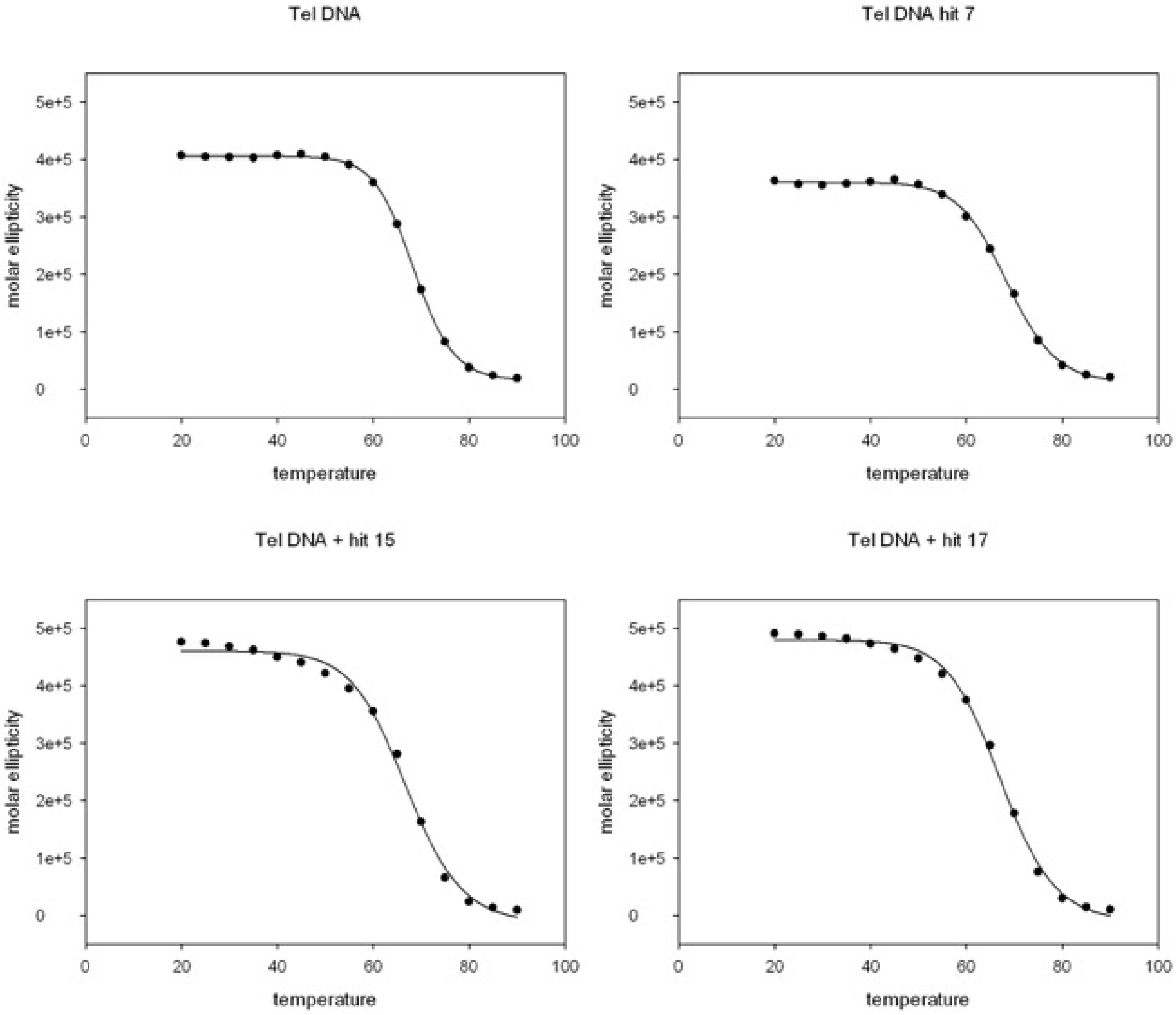

Figure S8. CD thermal unfolding analysis of Tel DNA G4 in complex with *hits 7*, ***15*** and ***17***. Melting curves of Tel G4 (4 μM) in the absence and presence of each hit (16 μM) plotted at the wavelength corresponding to the maximum CD signal.

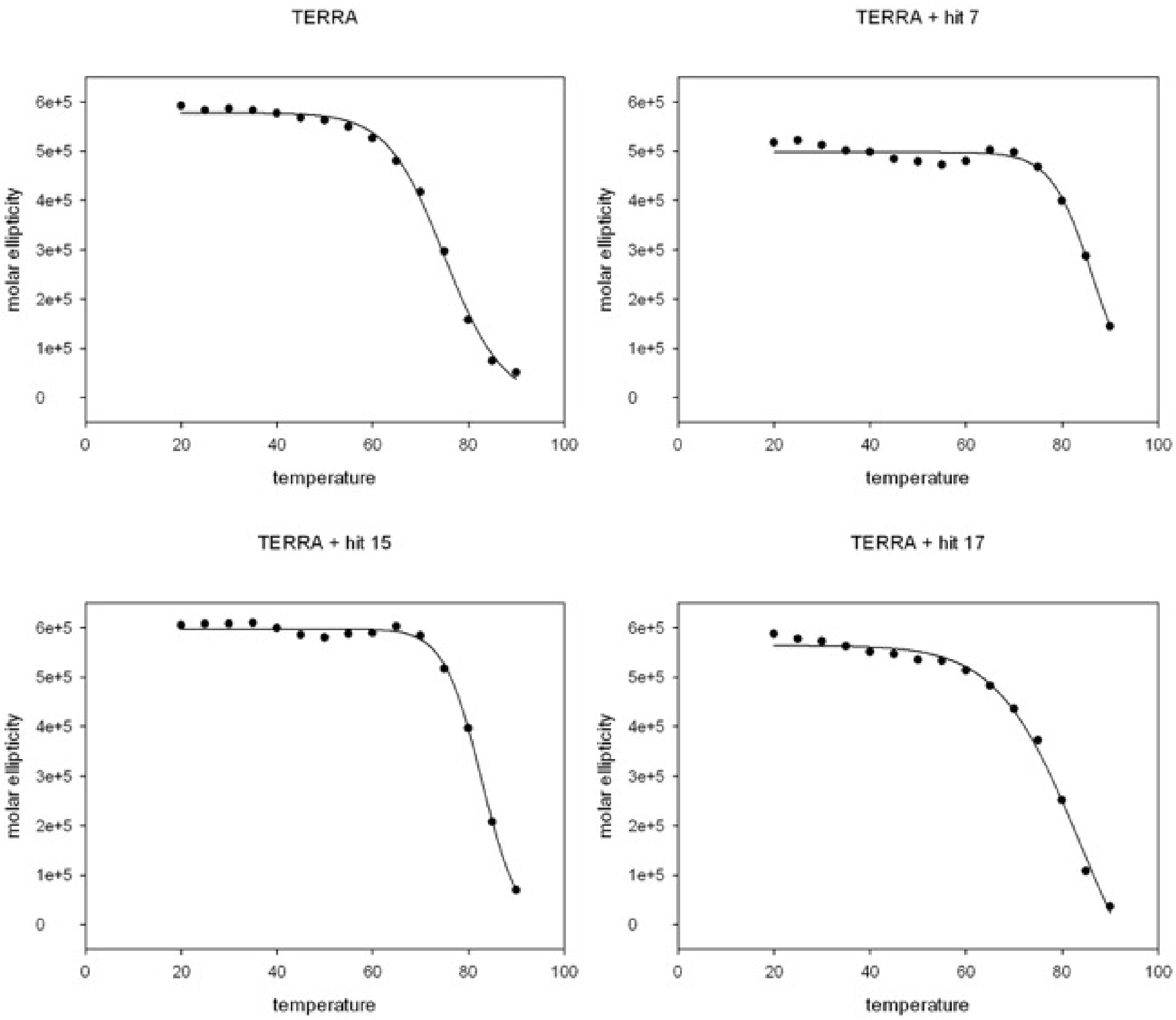

Figure S9. CD thermal unfolding analysis of TERRA G4 in complex with *hits* ***7***, ***15*** and ***17***. Melting curves of TERRA G4 (4 μM) in the absence and presence of each hit (16 μM) plotted at the wavelength corresponding to the maximum CD signal.

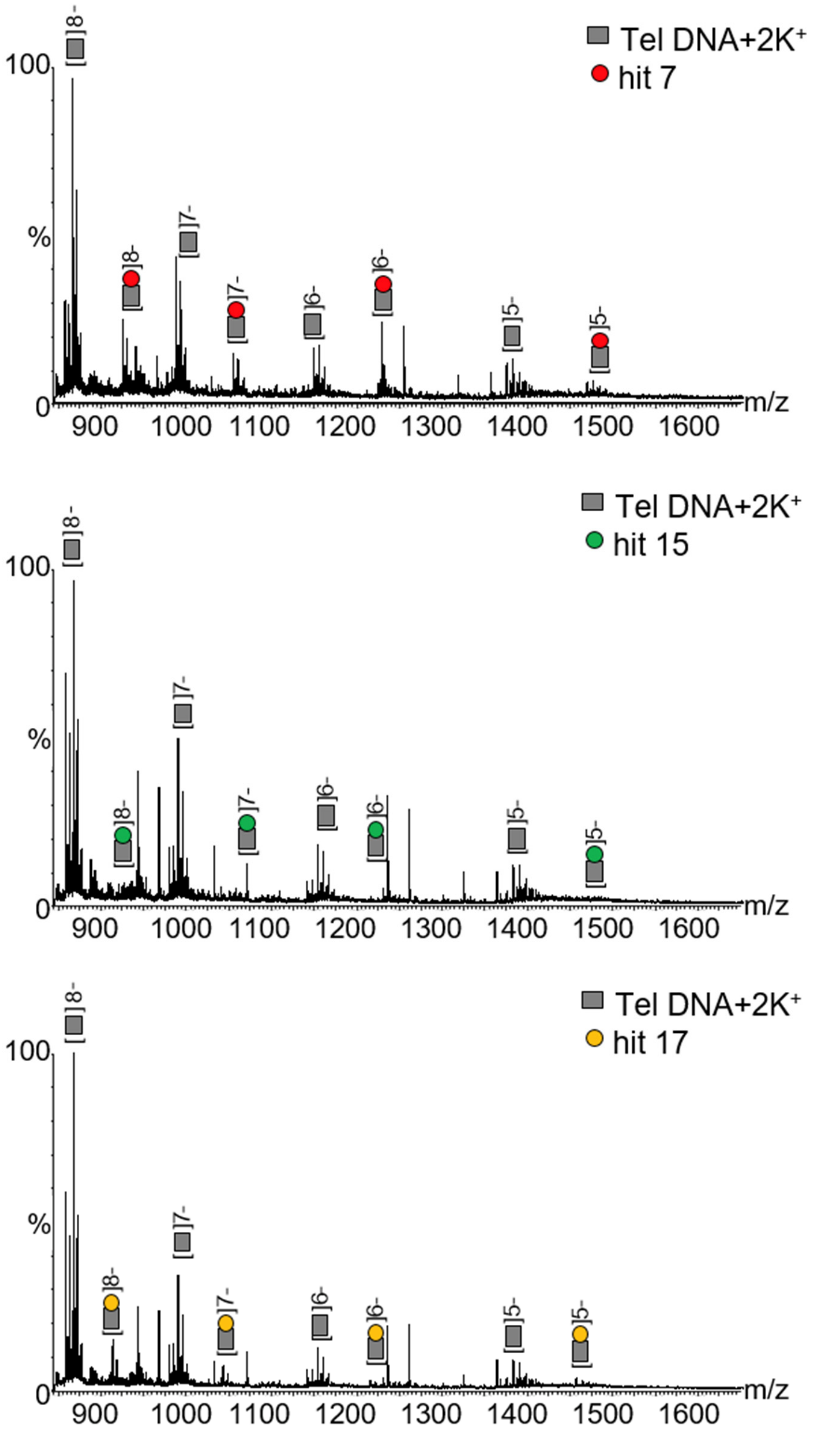

Figure S10. MS spectra of Tel (grey squares) incubated with the indicated hits. Samples containing Tel DNA oligonucleotide (5 µM) and hit molecule (10 µM) were incubated in MS buffer (HFIP 120 mM/TEA pH 7.4, KCl 0.8 mM, isopropanol 20%) overnight before MS analysis. The relevant m/z range is shown.

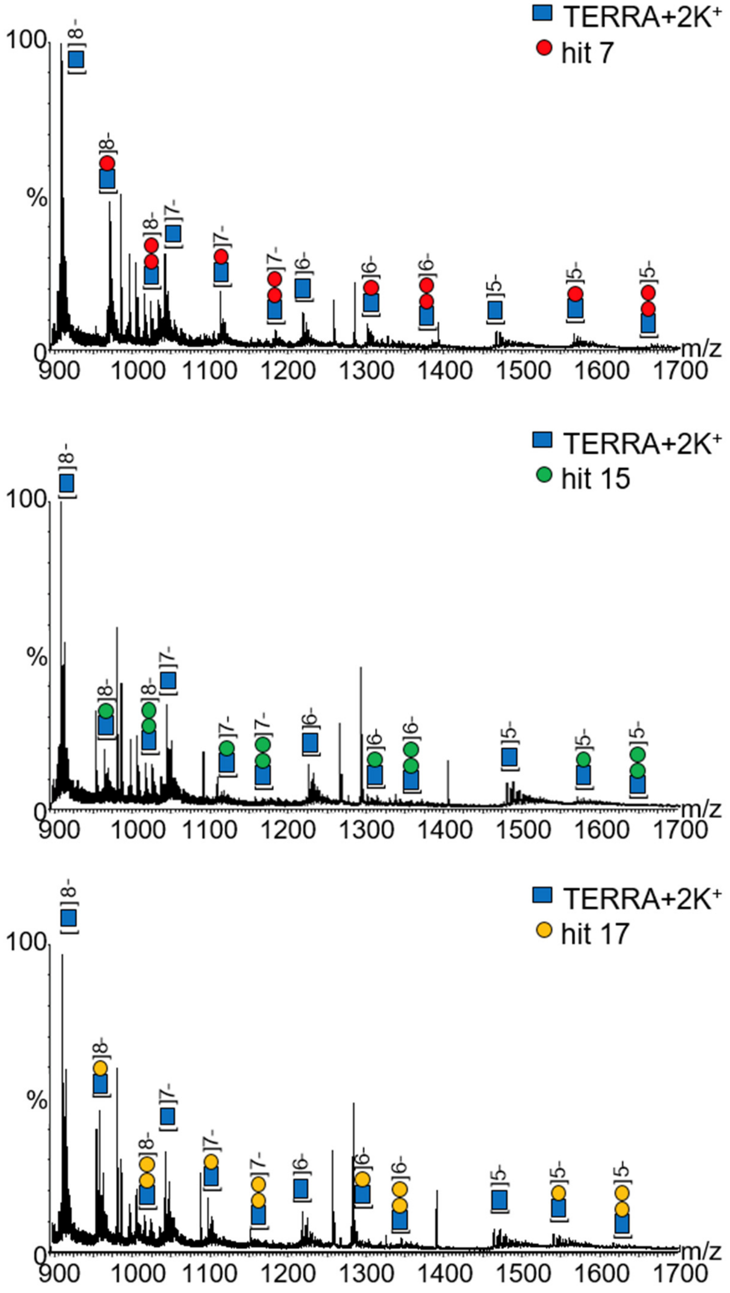

Figure S11. MS spectra of TERRA (blue squares) incubated with the indicated hits. Samples containing TERRA oligonucleotide (5 µM) and hit molecule (10 µM) were incubated in MS buffer (HFIP 120 mM/TEA pH 7.4, KCl 0.8 mM, isopropanol 20%) overnight before MS analysis. The relevant m/z range is shown.

Reference

1. Alcaro, S.; Costa, G.; Distinto, S.; Moraca, F.; Ortuso, F.; Parrotta, L.; Artese, A. The polymorphisms of DNA G-quadruplex investigated by docking experiments with telomestatin enantiomers. *Curr Pharm Des* **2012**, *18*, 1873-1879, doi:10.2174/138161212799958495.
